# A Meta-Analysis of Non-Osteoarthritis and Osteoarthritis Chondrocyte Gene Expression to Determine the Efficacy of Autologous Chondrocyte Transplantation as a Viable Treatment Option

**Published:** 2019-07-29

**Authors:** Tien M. Tran, Bryan Sosa, Alexis O’Connell, Tinchun Chu, Jessica A. Cottrell, Sulie L. Chang

**Affiliations:** 1 Department of Biological Sciences, College of Arts and Sciences, Seton Hall University, South Orange, New Jersey, USA; 2 Institute of NeuroImmune Pharmacology, Seton Hall University, South Orange, NJ USA

**Keywords:** Osteoarthritis, Autologous Chondrocyte Transplantation, Ingenuity Pathway Analysis

## Abstract

**Background:**

Osteoarthritis (OA) is a clinical syndrome characterized by joint failure that is accompanied by pain and functional limitations. OA is the leading cause of chronic disability in elderly and it is estimated that the United States spends $185 billion in management of OA annually. Although OA patients receive both pharmacologic and non-pharmacologic treatments, none of them provide long-lasting treatments. Since 1980s, autologous chondrocyte transplantation (ACT) has been used to regenerate cartilage within focal cartilage defects of young patients without pre-existing OA with increased functionality by 74% to 90%. In this technique, chondrocytes are removed from patients, multiplied in vitro, then implanted into the focal cartilage defect. Our review aimed to compare chondrocyte gene expression profiles of non-OA patients with OA patients to determine if OA-derived chondrocytes could be used for the ACT.

**Methods:**

An extensive literature search was conducted with following criteria:(1) comparing chondrocyte gene expression profiles of OA joint and non-OA joint, or (2)relating to ACT. Ingenuity Pathway Analysis (IPA) was then utilized to analyze the differential chondrocyte gene expression profiles of OA to non-OA patients to identify key associated biological pathways.

**Results:**

Differential gene expression profiles were similar between non-OA and OA chondrocytes: including ACAN, COL2A1, COL1A1, SOX 6 (p<0.001-0.05); FN1, COL11A1, MMP7, DLX5, SOX9, MMP2, TGFB1, THBS3, COMP, CILP2, ASPN, IGF2, DPT (p<0.001-0.05), and ADAMTS5, LAMA4 (p<0.01-0.05).

**Conclusion:**

These genes are important to cartilage function. Therefore, our results suggest that OA-derived chondrocytes may be useful to heal focal cartilage defects using ACT.

## Introduction

Osteoarthritis (OA) or degenerative joint disease, is a clinical syndrome characterized by joint failure that is accompanied by pain and functional limitation. There are many kinds of arthritis, however, OA is the most common form. It is also the leading cause of cause of pain and disability worldwide. As a frequent part of aging, if not inevitable, OA is the leading cause of chronic disability in the elderly [1].

Management of osteoarthritis is based on combination of medication and non-medication treatments, which aim to prevent the disease, modify the risk factors, and decrease the disease progression, improve tolerance for functional activity and quality of life [2]. Although OA patients receive both pharmacologic and non-pharmacologic treatments [3] pharmacologic options only relieve symptoms, while non-pharmacologic options such as arthroplasty only improve functionality temporarily. In the 1990s, a technique was developed by Brittberge and colleagues [4] called Autologous Chondrocyte Transplantation (ACT) which has been routinely used to regenerate cartilage within focal cartilage lesions of young patients without pre-existing OA [5]. In this technique, chondrocytes removed from patients are multiplied in vitro to achieve a minimum of 10 million cells and then are implanted into the focal cartilage defect. After transplantation, functionality increases by 74% to 90% [6]. The high improvement in function can be a potential treatment option for OA patients, who are still lacking a long-lasting treatment like ACT. Such promise encouraged us to conduct this review.

The purpose of this systematic review was to compare chondrocyte gene expression profiles of non-OA patients (before undergoing ACT) with OA patients (with or without arthroplasty) to determine if OA-derived chondrocytes could be used for the ACT technique.

Using Ingenuity Pathway Analysis (IPA) a powerful commercial software program we analyzed this data set and made meaningful interpretation of the gene expression data [7].

## Patients/Material and Methods

The systematic review was developed in accordance with the PRISMA statement.

### Data sources and Search strategy

A systematic literature search was conducted in electronic databased, including Quiagen knowledge base, PubMed, MEDLINE, New England Journal of Medicine (NEJM), EMBASE and Cochrane Library database, to obtain eligible studies. Key words and subject terms included “osteoarthritis”, “differential chondrocyte gene expression”, “autologous chondrocyte transplantation”, “aging-induced osteoarthritis”, and “non-osteoarthritic joint”. Initial search was done to collect an expansive number of research articles. Then, the titles and abstracts of the articles were used to apply the inclusion and exclusion criteria described as below.

### Inclusion and exclusion criteria

Articles or studies included in this review met the following two criteria: (1) the study focused on autologous chondrocyte transplantation and (2) there was a comparison data between gene expression profiles of osteoarthritic and non-osteoarthritic joints.

### Selection of studies

Duplicates were removed and relevant studies identified from the search selected for screenings. Studies were independently screened by their titles and abstracts by each reviewer. Then the full text was evaluated by the same authors. Disagreement between the two reviewers was resolved by consensus involving a third independent reviewer.

### Outcome measurement and data extraction

Two reviewers independently extracted data. In each eligible article/study, the information was extracted as follows: PMID, first author, year of publication, country in which the study was conducted, differential chondrocyte gene expression profile in OA joint and non-OA joint. A third reviewer was available for any assistance.

### Statistics

Data was input on each worksheet of Excel. Ingenuity Pathway Analysis (IPA) software was used to analyze the list of differential chondrocyte gene expression profiles in osteoarthritis patients versus non-osteoarthritis patients to identify the associated biological pathways.

## Results

### Studies identified

**Figure 1:**
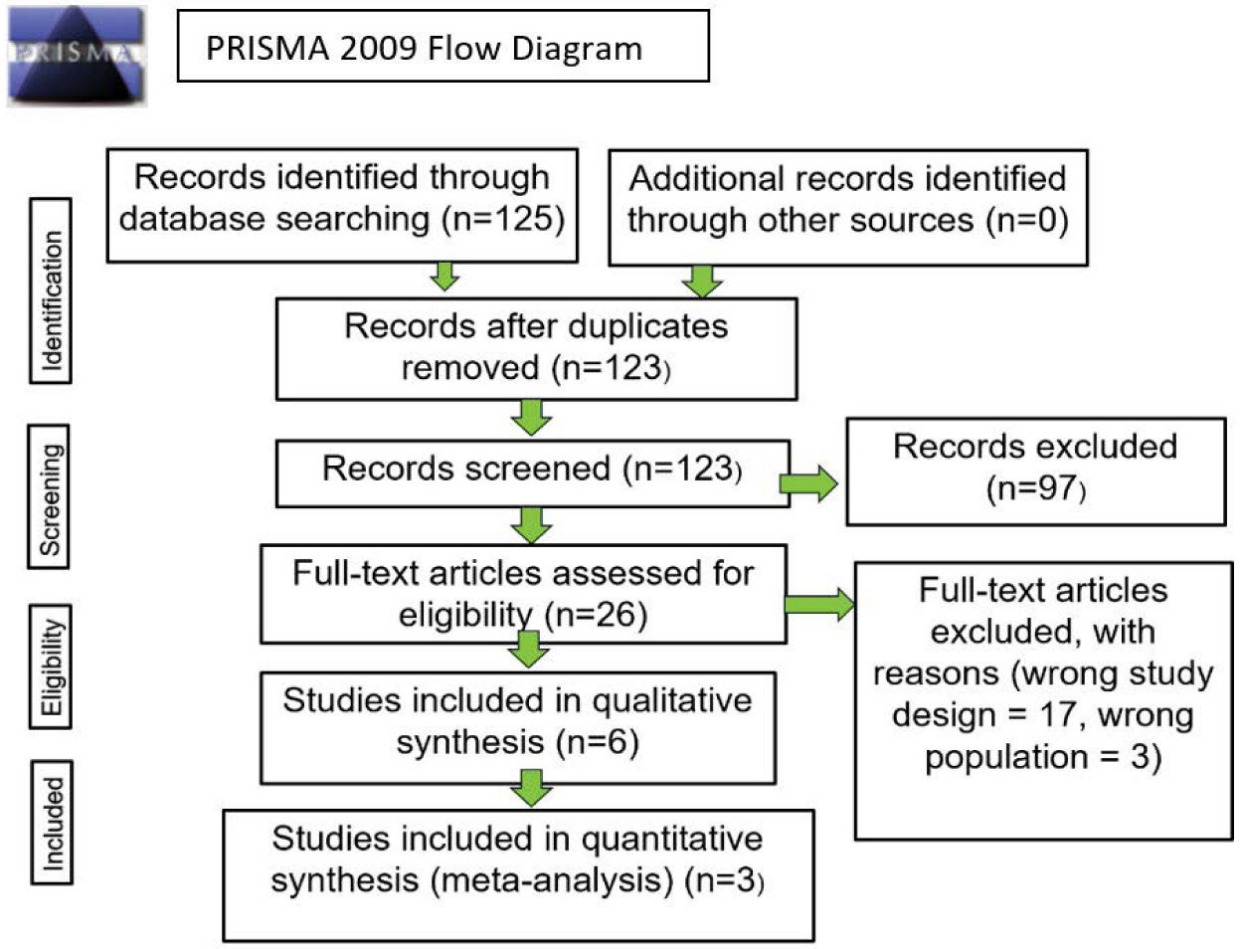
Flow-chart of Search Criteria used to identify included articles. Below shows the identification process for the articles used in this meta-analysis article

**Figure 2:**
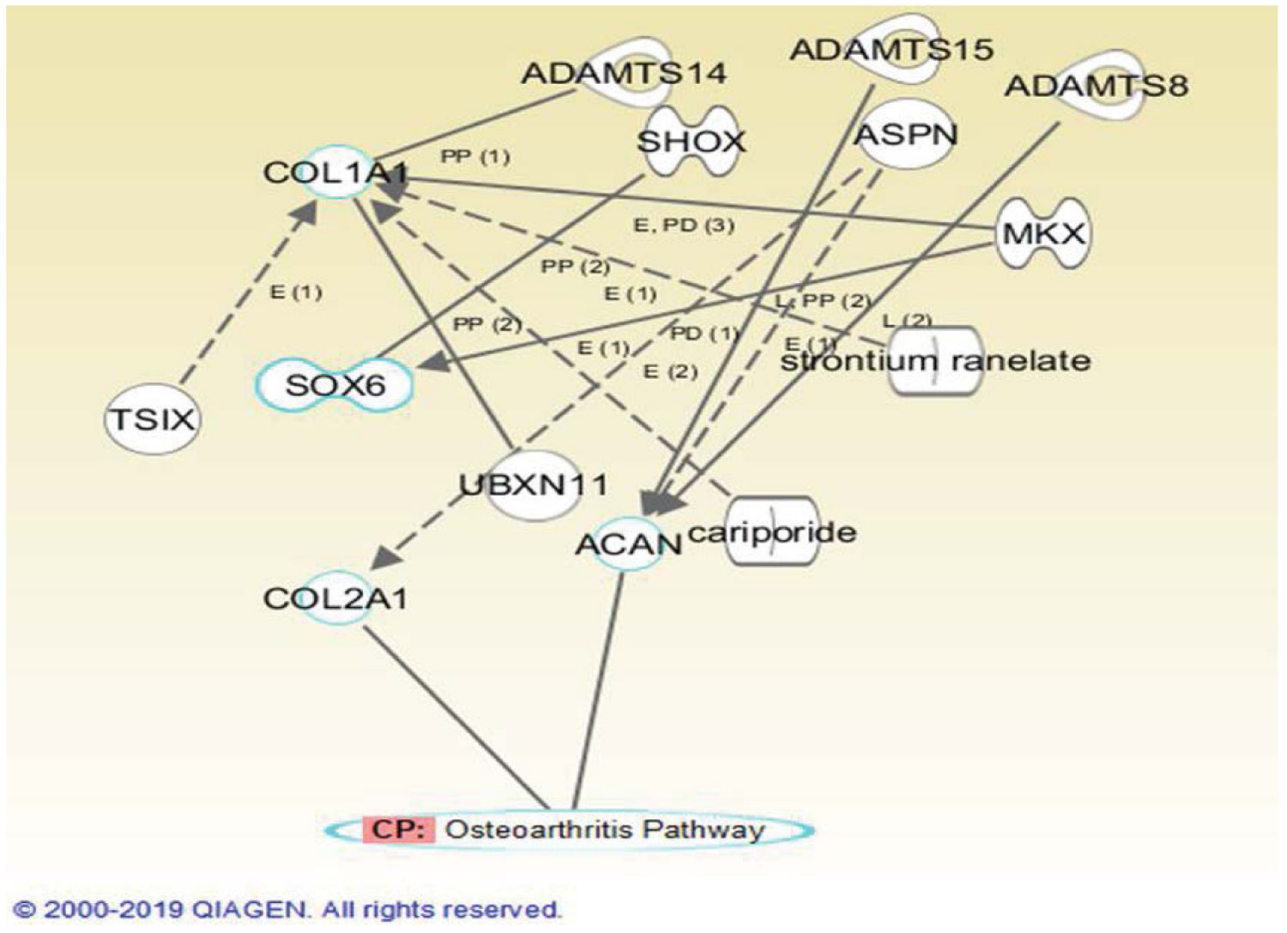
Depicted is the Osteoarthritis pathway in relation to the 4 identified genes. Genes that were found to be most relevant to the osteoarthritis pathway are highlighted in blue. Solid Lines represent a direct relationship while dashed lines represent an indirect relationship.

There were 151 studies identified through database searching. There was no additional record identified through other sources. After duplicates were removed, there were 125 studies identified by the search. This was followed by screening the titles and abstracts that led to the exclusion of 97 additional studies. The remaining 26 studies then were screened by full text for eligibility. There were 20 articles which were excluded with reasons such as inappropriate study design or population. Finally, there were 3 studies identified as eligible for meta-analysis. The number of studies identified and excluded at each stage is detailed in the Figure 1, Table 1. The characteristics of identified studies are provided in Table 2. Of the three included studies, the most recent study was 2016. The gene expression profile outcomes are the same in three studies including matrix proteins, and chondrogenic transcription factors. Two out of three studies also investigated the gene expression outcomes of hypertrophic or OA cartilage. When gene expression profiles of both OA patients and non-OA patients were compared differences in gene expression between each group were not found (Table 3 (p<0.001-0.05)). Of the four genes identified, three genes are important components of the extracellular matrix: aggrecan (ACAN), collagen 2a (COL2A), collagen 1 (COL1) and one gene is a transcription factor, SRY-box6 (SOX6). Utilizing Ingenuity Pathway Analysis (IPA), the four genes above were mapped to the osteoarthritis pathway to observe their connections to the pathway. The interaction is detailed in the Figure 2. COL2A1 and ACAN genes are directly associated with the Osteoarthritis pathway, which are represented by the solid lines, while COL1 and SOX6 are not, which are represented by the dotted lines. Gene expression profile in both OA patients and non-OA patients with increased expression with fold changes from 2 to 794 (p<0.001-0.05) are provided in Table 4. Out of thirteen genes identified, seven genes are extracellular matrix genes, one gene is cell adhesion, two genes are growth factors, two genes are transcription factors, and two genes are enzymes.

**Figure 3:**
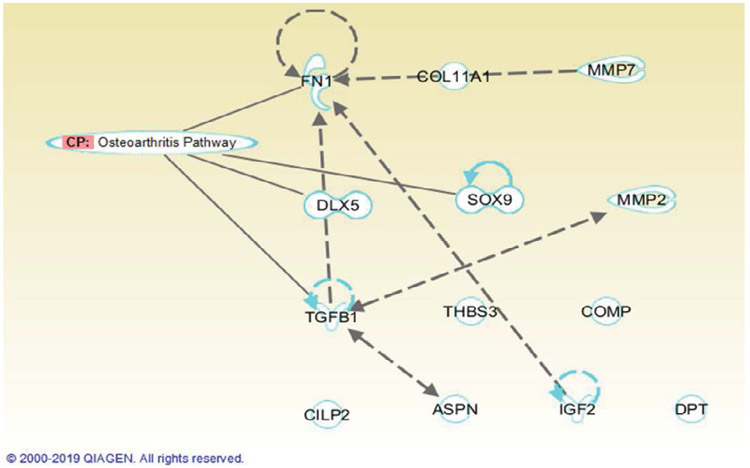
Depicted is the Relationship between the Osteoarthritis pathway and the 13 identified upregulated genes by IPA. Uprelated genes are highlighted in blue. Solid Lines represent a direct relationship while dashed lines represent an indirect relationship.

**Figure 4:**
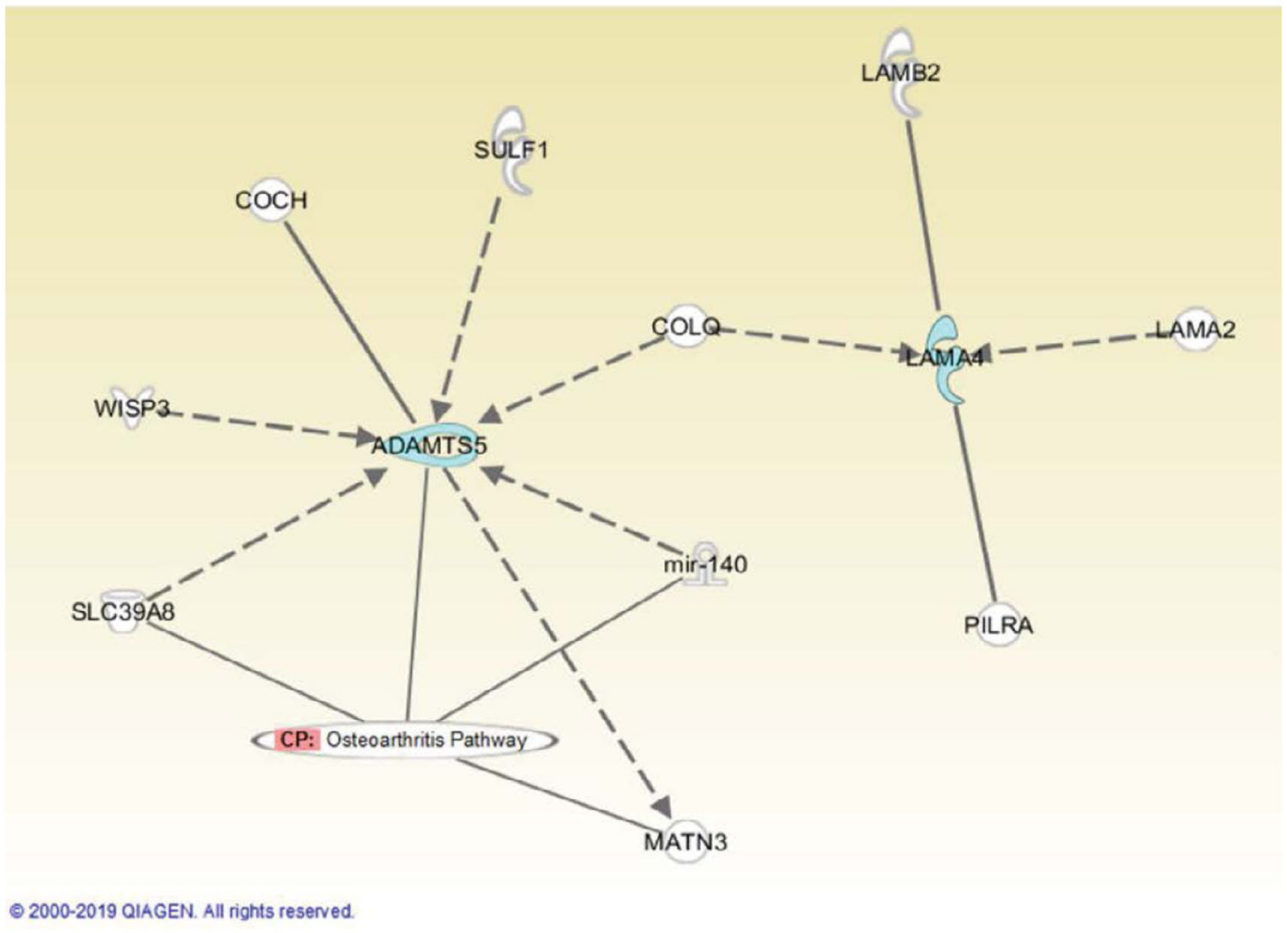
Depicted is the relationship between the Osteoarthritis pathway and the 2 identified genes which showed decreased in gene expression using IPA. Genes with decreased expression are highlighted in blue. Solid Lines represent a direct relationship while dashed lines represent an indirect relationship.

Utilizing Ingenuity Pathway Analysis, the thirteen genes with increased expression were mapped to the osteoarthritis pathway to observe their connections to the pathway. The interaction is detailed in the Figure 3. FN1, DLX5, SOX9 and TGFB1 genes are directly associated with the Osteoarthritis pathway, while COL11A1, MMP7, MMP2, THBS3, COMP, CILP2, ASPN, IGF2, DPT are not. Gene expression profile in both OA patients and non-OA patients with decreased expression with fold changes from −9 to −7 (p<0.01-0.05) are provided in Table 5. There is one gene of cell adhesion and receptors and one gene of transcription factors. Utilizing Ingenuity Pathway Analysis, the two genes with decreased expression were mapped to the osteoarthritis pathway to observe their connections to the pathway. The interaction is detailed in the Figure 4. ADAMTS5 has a direct connection with Osteoarthritis pathway while LAMA4 is not.

## Discussion

Although healthcare overall has improved, the management for the OA patients could be enhanced. Current treatments plans, target pain relief and the preservation of joint function using both pharmacological and non-pharmacological approaches. The ACT technique has been used since the 1990s to repair for focal cartilage defects in patients without pre-existing OA [7]. In order to apply this technique for patients with OA, it is crucial to determine whether the chondrocytes from OA patients have the same expression profile compared to chondrocytes from non-OA patients.

Based on this systematic review and meta-analysis of three published studies, we identified that there are four genes with similar expression patterns, thirteen genes with increased expression and three with decreased expression in OA-patients compared to non-OA patients in key genes associated with osteoarthritis.

The extracellular matrix genes are important for mature articular cartilage, including Aggrecan, CILP2, COL2A1, COL11A1, COMP, and FN1. The sign of chondrogenic differentiation was the increased expression of TGFB1 and DPT, which play important roles in increase the cellular response to TGFB [8]. The transcription factor SOX9 is considered a crucial factor in chondrogenesis and cartilage formation in vivo [9]. During chondrogenesis, SOX9 concomitantly binds to the sequences along the COL2A1 enhancer, the gene which encodes the type II collagen. Type II collagen is the important structural component of cartilage [10]. SOX 6 gene is also very important as it combines with SOX 9 to activate COL2A1, leading to a higher expression in OA chondrocytes. [11]. Both OA and non-OA chondrocytes showed gene expression of cartilage matrix components ACAN, CILP2, COL2A1, COL1, COL11A1, COMP, DPT, FN1, TIMP4, which meant chondrocytes derived from two groups of patients can have potential expression of matrix components for a hyaline cartilage phenotype.

Matrix metalloproteinase (MMP) composing of 16 different molecules which are involved in the degradation of extracellular matrix (ECM) under various pathological conditions [12]. Two of the MMP enzymes are the MMP 2 and MMP 7 (matrix metalloproteinase 2 and matrix metalloproteinase 7). These enzymes are capable to degrade different ECM components such as cartilage proteoglycan which is important for articular cartilage function. There are studies showed that MMP2, MMP7 gene increased expression in OA chondrocytes in the presence of interleukin-1 alpha and tumor necrosis factor alpha [12], which meant chondrocytes derived from OA increase expression of cytokine-induced MMP2 and MMP7. This is a part of the pathophysiology of osteoarthritis. In this study, we identified that MMP2, MMP7 genes increased expression in both OA patients and non-OA patients. That means patients without OA can increase the chance of have OA in the future as their chondrocytes increase expression of MMP2, MMP7 as chondrocytes derived from OA patients. Many studies have shown that MMPs are very critical molecules that control matrix remodeling occurring during the extracellular matrix (ECM) regeneration, and differentiation of MSCs to chondrocytes [13, 14]. ECM remodeling is an important process for skeletal formation in which chondrocytes progenitor cells undergo differentiation [15]. When there is an increase in collagen concentration, human MSCs increase the chondrogenic differentiation [16]. The degradation of articular cartilage is also affected by the other enzyme like ADAMTS5 [17]. This enzyme, together with metalloproteinases MMP promotes matrix degradation [18]. Cell adhesion gene LAMA4 (laminin alpha 4) is a constituent of basal membranes in blood vessels, the heart and the kidney. There are studies that show treating human chondrocyte cultures with a function-inhibiting antibody to LAMA4 can lead to a decrease in metalloproteinase gene, which will lead to decrease the ECM degradation. So decreased expression of LAMA4 and ADAMTS5 can decrease the degradation of ECM. In this study, we identified that there are decreased gene expression of ADAMTS5 and LAMA4. ADAMTS5 is responsible for aggrecan degradation which may play important role in cartilage destruction of mouse osteoarthritis model [19]LAMA4, in the other hand, contributes to clustering of human osteoarthritic chondrocytes which is a part of morphology in OA [20, 21] Using IPA, it showed the protein interaction network of ADAMTS 5, LAMA4 and co-expression gene (Figure 4).

**Figure 5:**
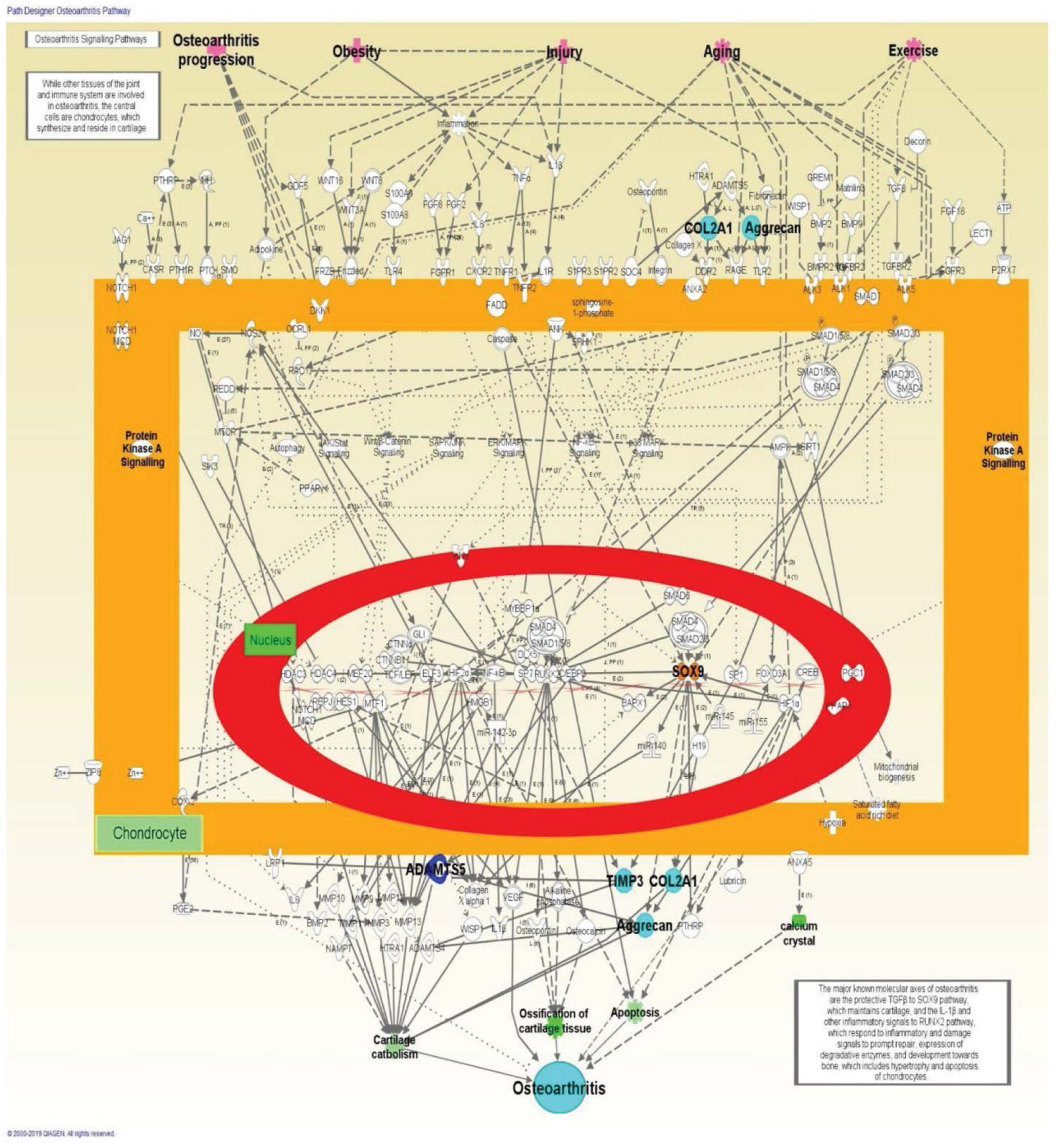
The canonical osteoarthritis pathway in relation to identified genes with same expression, increased expression, decreased expression. Genes that are upregulated are highlighted in light blue and those that are downregulated are found in dark blue.

Using IPA, the canonical osteoarthritis pathway is observed at a larger scope as detailed in the Figure 5. In the pathway is noticed that other health conditions, including obesity, injury, aging, exercise, and progressive osteoarthritis can lead to osteoarthritis.

Overall, based on our review, the data showed that gene expression profiles are similar between non-OA and OA chondrocytes for many genes with important cartilage formation functions. Our findings suggest that OA-derived chondrocytes may be useful to replace cartilage in defects using the ACT technique.

Gene expression profiles of chondrocytes from OA donors are similar to from non-OA donors, especially genes important for cartilage formation and functions. Patients without OA can increase the chance of have OA in the future as their chondrocytes have increased expression of MMP2 and MMP7 which is associated with the development of OA. In addition, other health conditions, including obesity, injury, aging, exercise, and progressive osteoarthritis can lead to osteoarthritis.

## Figures and Tables

**Table 1:** PRISMA checklist items used in this review

Section/topic	#	Checklist item	Reported on page #
**TITLE**			
Title	1	Identify the report as a systematic review, meta-analysis, or both.	1
**ABSTRACT**			
Structured summary	2	Provide a structured summary including, as applicable: background; objectives; data sources; study eligibility criteria, participants, and interventions; study appraisal and synthesis methods; results; limitations; conclusions and implications of key findings; systematic review registration number.	2
**INTRODUCTION**			
Rationale	3	Describe the rationale for the review in the context of what is already known.	3
Objectives	4	Provide an explicit statement of questions being addressed with reference to participants, interventions, comparisons, outcomes, and study design (PICOS).	3
**METHODS**			
Protocol and registration	5	Indicate if a review protocol exists, if and where it can be accessed (e.g., Web address), and, if available, provide registration information including registration number.	4
Eligibility criteria	6	Specify study characteristics (e.g., PICOS, length of follow-up) and report characteristics (e.g., years considered, language, publication status) used as criteria for eligibility, giving rationale.	4
Information sources	7	Describe all information sources (e.g., databases with dates of coverage, contact with study authors to identify additional studies) in the search and date last searched.	4
Search	8	Present full electronic search strategy for at least one database, including any limits used, such that it could be repeated.	4
Study selection	9	State the process for selecting studies (i.e., screening, eligibility, included in systematic review, and, if applicable, included in the meta-analysis).	4
Data collection process	10	Describe method of data extraction from reports (e.g., piloted forms, independently, in duplicate) and any processes for obtaining and confirming data from investigators.	4
Data items	11	List and define all variables for which data were sought (e.g., PICOS, funding sources) and any assumptions and simplifications made.	4
Risk of bias in individual studies	12	Describe methods used for assessing risk of bias of individual studies (including specification of whether this was done at the study or outcome level), and how this information is to be used in any data synthesis.	5
Summary measures	13	State the principal summary measures (e.g., risk ratio, difference in means).	5
Synthesis of results	14	Describe the methods of handling data and combining results of studies, if done, including measures of consistency (e.g., I^2^) for each meta-analysis.	5
Risk of bias across studies	15	Specify any assessment of risk of bias that may affect the cumulative evidence (e.g., publication bias, selective reporting within studies).	5
Additional analyses	16	Describe methods of additional analyses (e.g., sensitivity or subgroup analyses, meta-regression), if done, indicating which were pre-specified.	4,5
**RESULTS**			
Study selection	17	Give numbers of studies screened, assessed for eligibility, and included in the review, with reasons for exclusions at each stage, ideally with a flow diagram.	5
Study characteristics	18	For each study, present characteristics for which data were extracted (e.g., study size, PICOS, follow-up period) and provide the citations.	5
Risk of bias within studies	19	Present data on risk of bias of each study and, if available, any outcome level assessment (see item 12).	5
Results of individual studies	20	For all outcomes considered (benefits or harms), present, for each study: (a) simple summary data for each intervention group (b) effect estimates and confidence intervals, ideally with a forest plot.	8-12
Synthesis of results	21	Present results of each meta-analysis done, including confidence intervals and measures of consistency.	8-12
Risk of bias across studies	22	Present results of any assessment of risk of bias across studies (see Item 15).	8-12
Additional analysis	23	Give results of additional analyses, if done (e.g., sensitivity or subgroup analyses, meta-regression [see Item 16]).	8-12
**DISCUSSION**			
Summary of evidence	24	Summarize the main findings including the strength of evidence for each main outcome; consider their relevance to key groups (e.g., healthcare providers, users, and policy makers).	13-15
Limitations	25	Discuss limitations at study and outcome level (e.g., risk of bias), and at review-level (e.g., incomplete retrieval of identified research, reporting bias).	13-15
Conclusions	26	Provide a general interpretation of the results in the context of other evidence, and implications for future research.	13-15
**FUNDING**			
Funding	27	Describe sources of funding for the systematic review and other support (e.g., supply of data); role of funders for the systematic review.	16

**Table 2:** Characteristics of Each Study Used in the Meta-analysis

Author	Year	Country	Type of culture	Gene expression profile outcomes
**Lin et al.**	2008	Australia	Monolayer vs Cartilage tissue	Matrix proteins, enzyme proteases, cytokines, chondrogenic transcription factors
**Dehne et al.**	2009	Germany	Monolayer vs Scaffold	Extracellular matrix, cell adhesion and receptors, growth factors, chondrogenic transcription factors, enzyme proteases, hypertrophic or OA cartilage
**Vivek et al.**	2016	Austria	Monolayer	Matrix proteins, chondrogenic transcription factors, hypertrophic or OA cartilage

**Table 3:** Gene expression profiles of the below genes were found to be similar in both OA patients and non-OA patients

Gene symbol	Gene name
***Extracellular matrix***	
**ACAN**	Aggrecan
**COL2A**	Collagen type 2alpha
**COL1**	Collagen type 1
***Transcription factors***	
**SOX6**	SRY (sex determining region Y)-box6

**Table 4:** Gene expression profile in both OA patients and non-OA patients with increased expression

*Gene symbol*	Gene name	Fold changes
***Extracellular matrix***		
**ASPN**	Asporin	6.0-18.8
**CILP2**	Cartilage intermediate layer protein 2	71.3-78.2
**COL11A1**	Collagen type XI, alpha 1	5.9-10.4
**COMP**	Cartilage oligomeric matrix protein	128.0-794.2
**DPT**	Dermatopontin	44.2-69.7
**FN1**	Fibronectin 1	5.7-34.6
***Cell adhesion and receptors***		
**THBS3**	Thrombospondin 3	8.2-8.5
**Growth factors**		
**IGF2**	Insulin-like growth factor 2	43.5-114.9
**TGFB1**	Transforming growth factor beta 1	2.4-3.0
***Transcription factors***		
**DLX5**	Distal less homeobox	5.1-25.6
**SOX9**	SRY (sex determining region Y)-box 9	4.4-11.8
**Enzymes**		
**MMP2**	Matrix metalloproteinase 2	1.9-3.4
**MMP7**	Matrix metalloproteinase 7	107.2-109.7

**Table 5:** Gene expression profile in both OA patients and non-OA patients with decreased expression

Gene symbol	Gene name	Fold Changes
***Cell adhesion and receptors***		
**LAMA 4**	Laminin alpha 4	(−8.6) – (−6.6)
***Transcription factors***		
**ADAMTS5**	ADAM metalloproteinase with thrombospondin type 1 motif, 5	(−8.8) – (−7.6)

## References

[1] Bitton R (2009) The economic burden of osteoarthritis. Am J Manag Care 15:S230–S235. [View Article] 19817509

[2] Yu SP, DJ Hunter (2015) Managing osteoarthritis. Aust Prescr 38:115–119.[View Article] 26648637 10.18773/austprescr.2015.039 PMC4653978

[3] Zhang W, Moskowitz RW, Nuki G, Abramson S, Altman RD, (2007) OARSI recommendations for the management of hip and knee osteoarthritis, part I: critical appraisal of existing treatment guidelines and systematic review of current research evidence. Osteoarthritis Cartilage 15:981–1000. [View Article] 17719803 10.1016/j.joca.2007.06.014

[4] Brittberg M, Lindahl A, Nilsson A, Ohlsson C, Isaksson O (1994) Treatment of deep cartilage defects in the knee with autologous chondrocyte transplantation. N Engl J Med 331:889–895. [View Article] 8078550 10.1056/NEJM199410063311401

[5] Dehne T, Karlsson C, Ringe J, Sittinger M, Lindahl A (2009) Chondrogenic differentiation potential of osteoarthritic chondrocytes and their possible use in matrix-associated autologous chondrocyte transplantation. ArthritisRes Ther 11:R133. [View Article] 10.1186/ar2800 PMC2787268 19723327

[6] Brittberg M, Tallheden T, jogren-Jansson BS, Lindahl L Peterson A (2001) Autologous chondrocytes used for articular cartilage repair:an update. Clin Orthop Relat Res (391 Suppl):S337–348. [view Article] 11603717 10.1097/00003086-200110001-00031

[7] Krämer A, Green J, Pollard J, Tugendreich S (2014). Causal analysis approaches in Ingenuity Pathway Analysis. Bioinformatics 30: 523–530. [View Article] 24336805 10.1093/bioinformatics/btt703 PMC3928520

[8] Hayes AJ, MacPherson S, Morrison H, Dowthwaite G, Archer CW (2001) The development of articular cartilage: evidence for an appositional growth mechanism. Anat Embryol (Berl) 203:469–479. [View Article] 11453164 10.1007/s004290100178

[9] Lin Z, Fitzgerald JB, Xu J, Willers C, Wood D, (2008) Gene expression profiles of human chondrocytes during passaged monolayer cultivation. J Orthop Res 26:1230–1237. [View Article] 18404652 10.1002/jor.20523

[10] Bell DM, Leung KK, Wheatley SC, Ng LJ, Zhou S, (1997) SOX9 directly regulates the type-II collagen gene. Nat Genet 16:174–178. [View Article] 9171829 10.1038/ng0697-174

[11] Ikeda T, Kamekura S, Mabuchi A, Kou I, Seki S, Takato T, (2004). The combination of SOX5, SOX6, and SOX9 (the SOX trio) Arthritis Rheum. 50:3561–3573. [View Article] 15529345 10.1002/art.20611

[12] Ohta S, Imai K, Yamashita K, Matsumoto T, Azumano I, (1998) Expression of matrix metalloproteinase 7 (matrilysin) in human osteoarthritic cartilage. Lab Invest 78: 79–87. [View Article] 9461124

[13] Mott JD, Werb Z (2004) Regulation of matrix biology by matrix metalloproteinases. Curr Opin Cell Biol 16:558–564. [View Article] 15363807 10.1016/j.ceb.2004.07.010 PMC2775446

[14] Kasper G, Dankert N, Tuischer J, Hoeft M, Gaber T, (2007) Mesenchymal stem cells regulate angiogenesis according to their mechanical environment. Stem Cells 25:903–910. [View Article] 17218399 10.1634/stemcells.2006-0432

[15] Mannello F, Tonti GA, Bagnara GP, Papa S (2006) Role function of matrix metalloproteinases in the differentiation and biological characterization of mesenchymal stem cells. Stem Cells 24:475–481. [View Article] 16150919 10.1634/stemcells.2005-0333

[16] Hui TY, Cheung KM, Cheung WL, Chan D, Chan BP (2008) In vitro chondrogenic differentiation of human mesenchymal stem cells in collagen microspheres: influence of cell seeding density and collagen concentration. Biomaterials 29:3201–3212. [View Article] 18462789 10.1016/j.biomaterials.2008.04.001

[17] Fuerst FC, Gruber G, Stradner MH, Jones JC, Kremser ML, (2011) Regulation of MMP3 by laminin alpha 4 in human osteoarthritic cartilage. Scand J Rheumatol 40:494–496. [View Article] 22150225 10.3109/03009742.2011.605392 PMC4667954

[18] Echtermeyer F, Bertrand J, Dreier R, Meinecke I, Neugebauer K, (2009) Syndecan-4 regulates ADAMTS-5 activation and cartilage breakdown in osteoarthritis. Nat Med 15:1072–1076. [View Article] 19684582 10.1038/nm.1998

[19] Zhou X, Jiang L, Zhang Y, Zhang J, Zhou D, (2019) Genetic variation of aggrecanase-2 (ADAMTS5) in susceptibility to osteoarthritis. Braz J Med Biol Res 52:e8109. [View Article] 30652828 10.1590/1414-431X20188109 PMC6328970

[20] Moazedi-Fuerst FC, Gruber G, Stradner MH, Guidolin D, Jones JC, (2016) Effect of Laminin-A4 inhibition on cluster formation of human osteoarthritic chondrocytes. J Orthop Res 34:419–426. [View Article] 26295200 10.1002/jor.23036 PMC5727909

[21] Bennell KL, Hunter DJ, Hinman RS (2012) Management of osteoarthritis of the knee. Bmj 345:e4934. [View Article] 22846469 10.1136/bmj.e4934

